# Research on Wireless Passive Ultrasonic Thickness Measurement Technology Based on Pulse Compression Method

**DOI:** 10.3390/s24248023

**Published:** 2024-12-16

**Authors:** Long Pan, Kunsan Shi, Lei Han, Dingrong Qu, Yanling Zhang, Wenwu Chen

**Affiliations:** SINOPEC Research Institute of Safety Engineering Co., Ltd., Qingdao 266000, China

**Keywords:** wireless passive ultrasonic thickness measurement, fixed-point thickness measurement, corrosion detection, measurement error

## Abstract

Fixed-point thickness measurement is commonly used in corrosion detection within petrochemical enterprises, but it suffers from low detection efficiency for localized thinning, limitations regarding measurement locations, and high equipment costs due to insulation and cooling layers. To address these challenges, this paper introduces a wireless passive ultrasonic thickness measurement technique based on a pulse compression algorithm. The research methodology encompassed the development of mathematical and circuit models for single coil and wireless energy transmission, the proposal of a three-terminal wireless energy mutual coupling system, and the establishment of a finite element model simulating the ultrasonic body wave thickness measurement and wireless energy transmission system. An experimental setup was constructed to conduct thickness measurements on metal samples varying in thickness, shape, and material composition. The experimental findings revealed that the wireless ultrasonic echo signal, when processed using the pulse compression algorithm, achieved a thickness measurement accuracy approximately ten times superior to that of the untreated echo signal. This significant improvement in accuracy facilitates the high-density deployment of thickness measurement points in petrochemical applications.

## 1. Introduction

Non-destructive testing (NDT) technologies are crucial for assessing material integrity and structural safety without compromising the functionality of tested objects [1,2,3,4]. Among NDT applications, thickness measurement is paramount for equipment safety and maintenance, particularly in preventing incidents such as leakages and explosions [5]. Various techniques have been developed for this purpose, including radiographic [6], magnetic induction [7], eddy current [8], and ultrasonic measurements [9]. Ultrasonic thickness measurement has emerged as the preferred method due to its non-contact approach, high precision, wide applicability, and rapid execution [10,11,12].

However, traditional ultrasonic thickness measurement technologies face challenges in petrochemical environments, including noise interference, susceptibility to harsh conditions, accessibility issues, and complications in equipment setup and power supply. These limitations underscore the need for an efficient, precise, and cost-effective ultrasonic thickness measurement technology.

Various methods have been developed to enhance ultrasonic thickness measurement techniques and broaden their applications. Javad Abbaszadeh Bargoshadia et al. [13] used multiple ultrasonic probes to measure steel pipe thickness. Jacek Szelążek et al. [14] utilized piezoelectric transducers to analyze Lamb wave transit times in thick-walled tubes. Frederic B. Cegla et al. [15] proposed using waveguide structures for high-temperature measurements. Makiko Kobayashi et al. [16] developed flexible piezoelectric sensors for defect detection in composite materials. More recently, efforts have focused on enhancing the accuracy and convenience of ultrasonic thickness measurement technologies. Pierre Claude Ostiguy et al. [17] generated low-frequency Lamb waves for coating thickness measurements.

Ultrasonic testing technology has been widely used in NDT and structural health monitoring due to its non-invasive nature and high sensitivity. Scholars have conducted extensive research on ultrasonic testing and developed numerous sensors. However, these sensors’ traditional wired connections make the long-term monitoring of fixed points impractical, which has led to increasing interest in wireless solutions. David W. Greve et al. [18] first proposed using wireless power for Lamb wave sensors to detect defects. Their cylindrical three-coil system with magnetic cores successfully excited and received A0 and S0 Lamb wave modes, laying the foundation for combining wireless power transmission with ultrasonic wave propagation. However, Greve’s three-coil structure was too large and relatively inefficient in practical applications.

Chenghuan Zhong et al. [19] proposed a planar spiral three-coil structure that significantly reduced the overall sensor size. They modeled the three-coil system, studied energy transmission between the coils, and ultimately conducted Lamb wave defect detection using this novel three-coil structure. James S. Chilles et al. [20] built upon this planar spiral three-coil research by developing embedded sensors placed within carbon fiber composite materials, expanding the application scenarios.

Akinori Tamura et al. [21] extended the three-coil network to a five-coil network and placed the fabricated patches under insulation layers, achieving thickness measurements of aluminum-clad insulation layers. Subsequently, Akinori Tamura et al. [22] successfully implemented guided wave regional thickness measurement based on wireless power transmission using two sets of coils combined with Lamb wave regional thickness measurement methods, further expanding the applications of wireless ultrasonic transmission.

While these studies expanded ultrasonic applications by combining wireless power transmission with ultrasound, defect detection using ultrasonic guided waves only focused on identifying defect echoes in ultrasonic reflections, without stringent requirements for echo signal-to-noise ratio. Wireless power transmission inevitably reduces signal-to-noise ratio, but these studies paid little attention to signal processing under low signal-to-noise conditions. Therefore, this paper proposes using pulse compression algorithms to address this issue and further expand the applications of wireless-powered ultrasonic systems.

In petrochemical applications requiring high-density thickness measurements, wireless piezoelectric ultrasonic technology offers convenience and cost-effectiveness, but lacks accuracy. Integrating this with pulse compression technology, known for enhancing signal-to-noise ratios, results in a promising solution that balances accuracy, convenience, and cost-effectiveness. This paper systematically investigates the energy conversion mechanism of wireless passive ultrasonic thickness measurements, simulates ultrasound propagation characteristics in test samples, and explores the potential of pulse compression algorithms in improving signal-to-noise ratios. We conduct practical thickness measurement experiments on metal samples of varying materials, shapes, and thicknesses. Our results demonstrate significant advantages in the measurements’ accuracy and convenience, providing valuable insights for the application of this technology in petrochemical scenarios.

## 2. Materials and Methods

**Figure 1:**
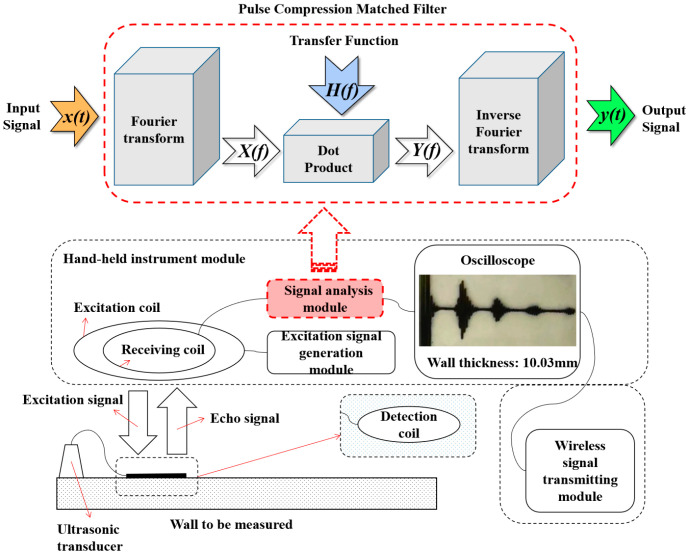
Ultrasonic thickness measurement based on wireless energy transmission.

As shown in Figure 1, the detailed process of the signal propagation pathway is as follows:

(1) The excitation signal generation module in the hand-held instrument generates an excitation signal with 5 cycles after linear frequency modulation and windowing processing, and then transmits it to the excitation coil.

(2) The excitation coil generates an excitation signal through electromagnetic induction and wirelessly transmits it to the detection coil in the form of a magnetic field.

(3) After receiving the excitation signal, the detection coil generates current by converting magnetism into electricity, which drives the crystals in the ultrasonic transducer to vibrate, thus generating ultrasonic waves that propagate into the object to be tested.

(4) When the ultrasonic waves travel from the upper surface to the lower surface of the object to be tested and then return to the upper surface again, they drive the ultrasonic transducer to vibrate again, generating current. This current then generates a magnetic field through the detection coil, and this magnetic field signal is called the echo signal.

(5) The receiving coil receives the echo signal by converting magnetism into electricity, and the current signal is transmitted to the signal analysis module.

(6) The signal analysis module is equipped with a pulse compression matched filter. Its working principle is to perform Fourier transforms on the received echo current signal and the excitation signal, respectively, and then conduct a point multiplication operation in the frequency domain (that is, to calculate the cross-correlation between the echo current signal and the excitation current signal). This operation is called “pulse compression processing”, which can effectively remove clutter interference and highlight the characteristics of the echo peak.

(7) The correlated waveform signal after pulse compression is calculated, using algorithms such as envelope detection and peak searching to obtain the time difference between the two echo peaks (this time difference is the time consumed by the ultrasonic waves traveling through the thickness path twice). Combined with the speed of sound, the thickness value can be calculated.

(8) The calculated thickness value is remotely transmitted to the analysis platform through the wireless transmission module to realize the comprehensive assessment of corrosion risks.

### 2.1. Theoretical Model

#### 2.1.1. Coil Model Development

**Figure 2:**
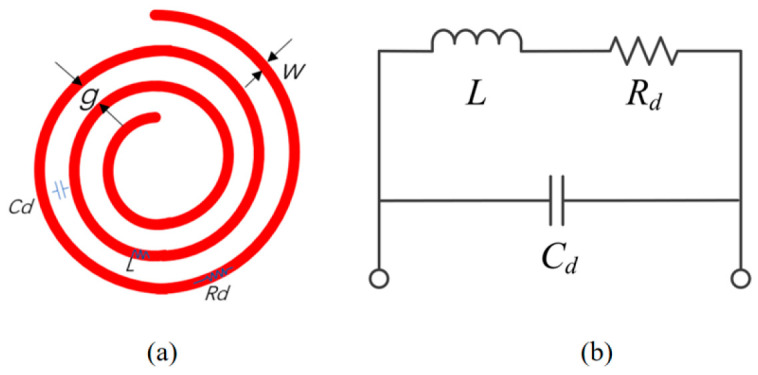
Coil models: (**a**) physical model, (**b**) circuit model.

To ensure both compactness and high transmission efficiency, the research adopts a planar spiral coil design, illustrated in Figure 2a. As shown in Figure 2b, the flat spiral coil is modeled as a parallel resonant circuit, which takes the electrical resistance of the wires and the electric fields that form between adjacent wires into consideration. The circuit includes an inductance *L*, a frequency-dependent resistance *R_d_*, and the parasitic capacitance *C_d_* between the turns of the coil. This configuration aims to optimize the coil’s design for effective energy transfer while minimizing its physical footprint.

#### 2.1.2. Coil Inductance Calculation

The inductance *L* of a coil largely depends on the coil’s physical parameters. In this study, considering factors such as cost, Printed Circuit Board (PCB) coils were employed for wireless energy transmission. The calculation of inductance for PCB coils is represented by Equation (1) [23], as follows:

(1) $$L=\frac{\mu_{0}N^{2}d_{avg}}{2}\left[\ln\left(\frac{2.46}{p}\right)+0.2p^{2}\right],$$

where *ρ* represents the fill factor, calculated as *p* = (*d_out_* − *d_in_*)/(*d_out_* + *d_in_*), where *d_out_* and *d_in_* are the outer and inner diameters of the coil, respectively. The average diameter *d_avg_* is given by *d_avg_* = (*d_out_* + *d_in_*)/2. *N* refers to the number of coil turns, while *d_out_* and *d_in_* represent the outer diameter and inner diameter of the coil, respectively. 

#### 2.1.3. Coil Resistance Calculation

Given the influence of the skin effect, the resistance of a PCB coil with a rectangular cross-section is calculated using Equation (2) [24], as follows:

(2) $$R_{d}=\frac{\rho_{c}l}{wh}\frac{h}{\delta \left(1-e^{-h/\delta }\right)(1+h/w)}\approx \frac{\rho_{c}l}{wh}\frac{h}{\delta \left(1-e^{-h/\delta }\right)},$$

where *ρ_c_* denotes the resistivity of the coil material with a value of 1.7 × $10^{-8\;}\Omega \cdot m$, *l* represents the total length of the coil $l=2\pi \overset{\underset{N}{i=1}}{\sum }a_{i}=\frac{\pi (d_{out}+d_{in})N}{2}$, *δ* is the skin depth calculated as $\sqrt{\frac{\rho }{\pi f\mu }}$, *f* is the current frequency, *μ_c_* is the magnetic permeability of the metal, *d_out_* and *d_in_* are the outer and inner diameters of the coil, respectively, and N refers to the number of turns in the coil. It is important to note that Equation (3) is only applicable when the condition *w* ≫ *h*, where *w* is the width and *h* is the height of the coil’s cross-section.

#### 2.1.4. Coil Capacitance Calculation

**Figure 3:**
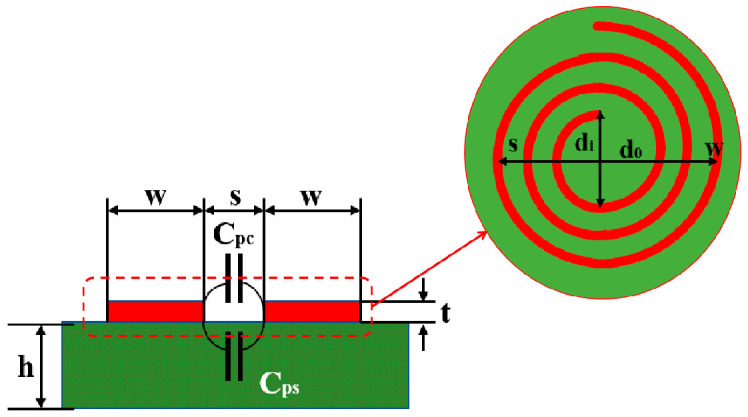
Circular PCB coil structure.

The simplified model of the internal structure of a PCB coil, as shown in Figure 3, illustrates key geometrical aspects relevant to capacitance calculation. In this model, *s* represents the radial spacing between turns, *t* denotes the track width, and *l_s_* (the total length of the spacing between lines) can be considered the length of the plates in a parallel plate capacitor. Under these conditions, the capacitance reflects the distributed capacitance of the coil, which is crucial for determining the coil’s performance in resonant circuits, especially for high-frequency applications. The expression for this capacitance is given by Equation (3) [23], as follows:

(3) $$C_{p}=C_{pc}+C_{ps}\approx \left(\alpha \varepsilon_{c}+\beta \varepsilon_{s}\right)\varepsilon_{0}\frac{t}{s}l_{s}\;l_{s}=\frac{\pi (d_{out}+d_{in})(N-1)}{2},$$

where *ε_c_* represents the relative permittivity of the filler material used in the PCB, while *ε_s_* denotes the relative permittivity of the substrate material. The coefficients *α* and *β* are proportionality factors that adjust for variations in the geometrical and material properties affecting the capacitance.

#### 2.1.5. Wireless Energy Transmission System Modeling

A comprehensive circuit model has been developed for the three-port wireless energy coupling system. The corresponding mathematical model can be represented by the impedance matrix shown in Equation (4) [19], as follows:

(4) $$\left[\begin{array}{c} V_{1}\\ V_{2}\\ V_{3} \end{array}\right]=\left[\begin{array}{c} Z_{11}Z_{12}Z_{13}\\ Z_{12}Z_{22}Z_{23}\\ Z_{13}Z_{23}Z_{33} \end{array}\right]\left[\begin{array}{c} I_{1}\\ I_{2}\\ I_{3} \end{array}\right]=\left[\begin{array}{ccc} R_{1}+j\omega L_{1} & j\omega M_{12} & j\omega M_{13}\\ j\omega M_{12} & R_{2}+j\omega L_{2} & j\omega M_{23}\\ j\omega M_{13} & j\omega M_{23} & R_{3}+j\omega L_{3} \end{array}\right]\left[\begin{array}{c} I_{1}\\ I_{2}\\ I_{3} \end{array}\right],$$

where *R_i_* represents the resistance of the coil, *L_i_* denotes the inductance of the coil, *M_ij_* is the mutual inductance between coils *i* and *j*, *Z_ij_* represents the mutual impedance between coils *i* and *j*, *w* is the angular frequency of the coil, and *Z_ii_* signifies the self-impedance of coil *i*.

**Figure 4:**
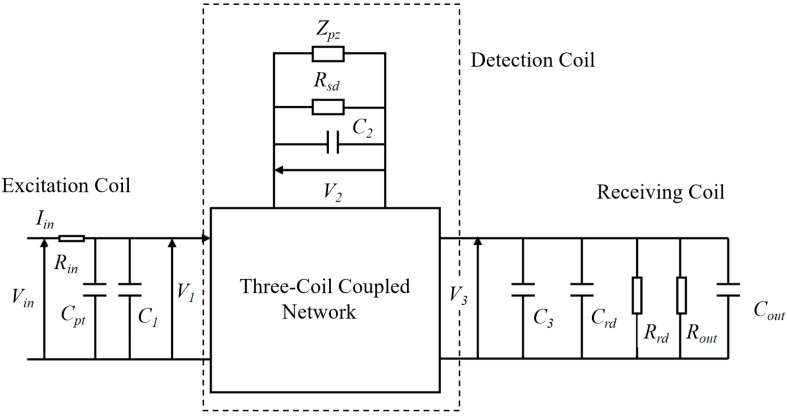
Three-coil coupled circuit model.

Figure 4 illustrates a refined electrical circuit network of mutual inductance for the three-port wireless energy transmission system. To enhance the model’s accuracy, additional electrical parameters beyond the aforementioned inductance and resistance have been incorporated into the circuit network. These include the following: input resistance (*R_in_*), output capacitance (*C_out_*), and output resistance (*R_out_*). Moreover, to enhance ultrasonic performance, several tuning elements were incorporated into the overall circuit for impedance matching. These include tuning capacitors *(C_pt_* and *C_rt_*) and tuning resistors (*R_sd_*, *R_rd_*).

Impedance matching, a critical aspect in electronic system design, involves the adjustment of input and output impedances to optimize system performance. This technique primarily serves two purposes: maximizing power transmission or minimizing signal reflection. In wireless transmission systems, proper impedance matching between Radio Frequency (RF) transmission equipment and receiving antennas is fundamental to achieving maximum power transfer efficiency.

To systematically analyze and optimize the impedance matching in our system, we established a comprehensive circuit model, as expressed in Equation (4). The analysis process involves a sequential transformation approach: first converting the three-circuit model to a two-circuit model, and subsequently to a single-circuit model. The corresponding input impedance calculations are mathematically represented in Equations (5) and (6), as follows. This methodical transformation enables precise impedance matching calculations, ultimately leading to optimized power transfer in the system.

(5) $$\left[\begin{array}{c} V_{1}\\ V_{3} \end{array}\right]=\left[\begin{array}{cc} Z_{11}-Z_{12}^{2}/\left(Z_{S}+Z_{22}\right) & Z_{13}-Z_{12}\cdot Z_{23}/\left(Z_{S}+Z_{22}\right)\\ Z_{13}-Z_{12}\cdot Z_{23}/\left(Z_{S}+Z_{22}\right) & Z_{33}-Z_{23}^{2}/\left(Z_{S}-Z_{22}\right) \end{array}\right]\left[\begin{array}{c} I_{1}\\ I_{3} \end{array}\right]$$

By transforming the three-circuit model into a single-circuit model through the above equation, and subsequently referring the secondary circuit elements to the primary side of the two-circuit model, we can derive the input impedance. The derived expression for the input impedance is given by the following:

(6) $$Z_{IN}=\frac{V_{in}}{I_{in}}=Z_{Rout}+\frac{1}{1/Z_{P}+1/Z_{C1}+1/Z_{Cpt}}$$

In the formula, $Z_{Rout}$ refers to the impedance of the output terminal resistance, $Z_{p}$ refers to the total impedance of the detection coil and the receiving coil, and $Z_{C1}$ and $Z_{Cpt}$ respectively refer to the impedances of the two tuning capacitors. 

#### 2.1.6. Signal Post-Processing Algorithm Based on Pulse Compression Algorithm

The pulse compression algorithm is used to transmit a wide pulse signal and compress it into narrow pulses through a specific filter at the receiving port, thereby improving the distance resolution of the ultrasound system while maintaining a sufficient detection distance. After pulse compression correlation calculation, the received signal can effectively identify the echo signal without reducing the distance resolution while increasing the signal distance.

The process of pulse compression signal is shown in Equation (6), assuming that the input signal is *x*(*t*) = *s*(*t*) + *n*(*t*), where *s*(*t*) is the effective signal and *n*(*t*) is the noise. The spectral density function of the effective signal is *S*(*f*); *y*(*t*) *= s*_0_(*t*) *+ n*_0_(*t*), *y*(*t*) is the sum of the output effective signal and noise, where *s*_0_(*t*) is the input effective signal processed by the pulse compression method. *H*(*f*) is the transfer function of the matched filter. This process can be represented by the following equation:

(7) $$s_{0}(t)=\int_{-\infty }^{+\infty }H(f)S(f)e^{j2\pi ft}df$$

The noise power spectral density of the processed signal can be derived by integrating the product of the input noise power spectral density and the squared magnitude of the matched filter’s transfer function across the frequency domain. The resultant noise power expression is mathematically represented as follows:

(8) $$N_{o}=\frac{n_{o}}{2}\int_{-\infty }^{+\infty }|H(f){|}^{2}df$$

By applying the Cauchy–Schwarz inequality, we obtain the following:

(9) $${\left|\int_{-\infty }^{+\infty }f_{1}(x)f_{2}(x)dx\right|}^{2}\leq \int_{-\infty }^{+\infty }{\left|f_{1}(x)\right|}^{2}dx\cdot \int_{-\infty }^{+\infty }{\left|f_{2}(x)\right|}^{2}dx$$

The equality condition for the above inequality is satisfied if $f_{1}(x)={kf}_{2}^{*}\left(x\right)$, where k represents an arbitrary constant.

By letting $f_{1}\left(x\right)=H\left(f\right)$, $f_{2}\left(x\right)=S\left(f\right)e^{j2\pi ft}$, we obtain the following:

(10) $$r\leq \frac{\int_{-\infty }^{+\infty }|H(f){|}^{2}df\cdot \int_{-\infty }^{+\infty }|S(f){|}^{2}df}{\frac{n_{o}}{2}\int_{-\infty }^{+\infty }|H(f){|}^{2}df}=\frac{2E}{n_{0}}$$

In this expression, where *E* denotes the energy of $s\left(t\right)$, the equality condition in Equation (10) is satisfied if $H\left(f\right)$ satisfies the following conditions corresponding to the maximum signal-to-noise ratio of the output signal:

(11) $$H(f)=kS^{*}(f)e^{-j2\pi ft}$$

The transmission characteristics $H\left(f\right)$ can alternatively be represented by its impulse response $h\left(t\right)$, expressed as follows:

(12) $$h(t)=(\frac{1}{2\pi })\int_{-\infty }^{\infty }H(\;f\;)e^{-j2\pi ft}df=Ks(t_{0}-t)$$

The processed output waveform can be mathematically represented by the following expression:

(13) $$s_{o}\left(t\right)=\int_{-\infty }^{\infty }s\left(t-\tau \right)h\left(\tau \right)d\tau =K\int_{-\infty }^{\infty }s\left(t-\tau \right)s\left(t_{0}-\tau \right)d\tau =KR\left({t-t}_{0}\right)$$

The numerical calculation process described above represents the pulse compression technique. From this analysis, we can conclude that the fundamental principle of pulse compression is to perform cross-correlation between the received signal and the excitation signal, producing an output that indicates their degree of matching. This process preserves the arrival time of echo signals in thickness measurement applications while effectively extracting echo signals from noise. Consequently, it significantly enhances the signal-to-noise ratio of the received signals, facilitating more accurate thickness calculations of the test specimen.

### 2.2. Data Simulation

#### 2.2.1. Simulation of the Ultrasonic Wave Propagation Process

To improve the accuracy of reproducing the ultrasonic wave propagation process, this study utilizes COMSOL Multiphysics to simulate the actual transduction process of piezoelectric materials.

**Figure 5:**
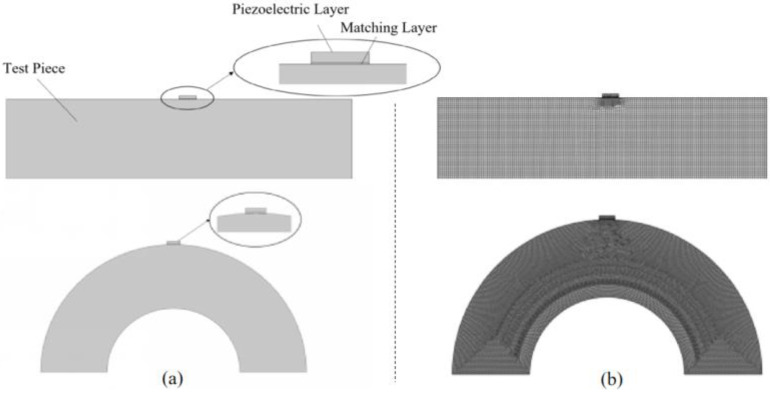
Two-dimensional ultrasonic body wave thickness measurement model. (**a**) Simulation modeling of the sensor and experimental sample (**b**) Meshing of the simulation model. The model consists of three layers from top to bottom, including the piezoelectric layer, the matching layer, and the specimen to be tested. In terms of specific physical field settings, the “Solid Mechanics” field was applied to all three parts, while “Electrostatics” was applied exclusively to the piezoelectric layer.

First, establish the plate specimen and pipe specimen as shown in Figure 5a using COMSOL’s “Geometry” command, and create two rectangles on them to simulate the piezoelectric element and matching layer. In COMSOL, set the materials for the piezoelectric element, matching layer, and test specimen as “Lead Zirconate Titanate”, “Acrylic plastic”, and “Aluminum”, respectively. The matching layer facilitates better transmission of ultrasonic waves from the piezoelectric element into the test specimen, with “Acrylic plastic” being one of the commonly used coupling agents.

Next, add “Electrostatics” and “Circuit” physics fields for the piezoelectric element, and “Solid Mechanics” physics field for the matching layer and test specimen. In the “Electrostatics” physics field, set “Terminal” and “Ground” conditions on the upper and lower surfaces of the piezoelectric element, establishing the potential difference through these boundary conditions. In the “Solid Mechanics” physics field, simply set “Low-Reflecting Boundary” conditions. Complete the physics field settings by adding “Voltage Source”, “Resistor”, and other conditions in the “Circuit” physics field. Then, select “Piezoelectric Effect” in the Multiphysics commands and apply it to the piezoelectric element to complete the setup.

For mesh settings, apply the “Swept” mesh for the plate specimen model and the “Free Tetrahedral” mesh for the pipe specimen model. The Free Tetrahedral mesh is necessary for the pipe specimen model because the connection between the matching layer and the pipe is curved rather than planar, allowing for better representation of the actual specimen conditions. Set the mesh size to the commonly used one-fifth wavelength to complete the model configuration.

Simultaneously, the required ultrasonic waves are generated through the piezoelectric effect. To simulate the propagation characteristics of ultrasonic waves within the specimen, a corresponding two-dimensional ultrasonic bulk wave thickness measurement model was established. The structure of the model and its mesh division are shown in Figure 5a,b, respectively.

For mesh generation, structured meshes were used for the piezoelectric layer, matching layer, and specimen areas. The mapped mesh sizes were set to 0.1 mm, 0.03 mm, and 0.5 mm, respectively. The maximum computational time step was set to 1 μs.

#### 2.2.2. Simulation of Parametric Scanning for Key Variable Parameters of the Coil

Establishing the Wireless Power Transfer System in COMSOL using the following directions:

First, create two coils by selecting “Helix” in the “Geometry” module and setting “Axial Pitch” to 0 to obtain planar spiral coils. Connect the coils using cylindrical “bridges” and establish a square region to define the air domain. Define the physics field as “Magnetic Field” and apply “Magnetic Field” physics along with “Coil” and “Geometric Analysis” commands to both coils. For material properties, set the coil material as “Copper” and the square region as “Air”, with the excitation condition being a three-cycle sinusoidal voltage. For the study steps, use transient settings, first adding the “Coil Geometric Analysis” command to be computed before the transient study. Determine optimal coil design parameters through parametric sweeps of coil turns and outer diameter.

**Figure 6:**
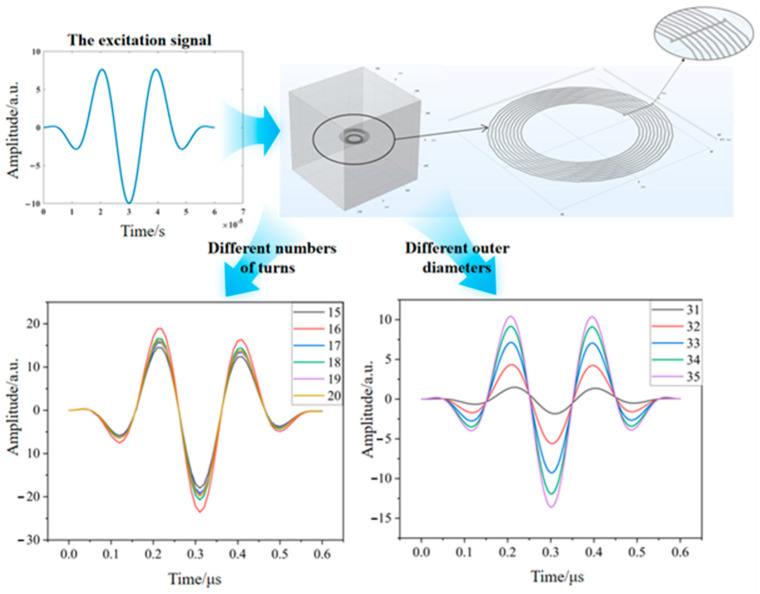
Coil parameterized scanning simulation diagram.

Taking the excitation coil as an example, perform parametric sweeps of its outer diameter and number of turns. The following Figure 6 shows the energy magnitude on the transducer coil under different parameters. As observed, maximum energy is achieved on the transducer coil when the excitation coil has an outer diameter of 35 mm and 16 turns.

Similarly, the specific parameters for the receiving coil and detection coil can be determined using this approach.

### 2.3. Experiment

To investigate the practical effectiveness of a wireless ultrasonic thickness measurement method based on wireless power transfer (WPT), we prepared plate specimens of varying thicknesses under laboratory conditions and constructed a wireless and passive ultrasonic thickness measurement system with a Tiepie-HS5 (manufactured by TiePie Engineering, Sneek, The Netherlands) and a computer as the core. By measuring the thickness of these plates, the good performance of the system was validated. In addition, we proposed signal processing methods to optimize measurement accuracy, further enhancing the overall system precision and reducing errors.

**Figure 7:**
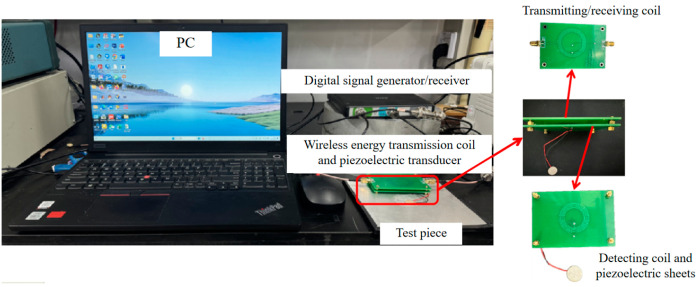
Test system composition.

Figure 7 illustrates the actual image of the experimental system. The system primarily consists of a Tiepie-HS5 signal generator and receiver, a laptop, a wireless ultrasonic transducer with integrated WPT capability, and the test specimens.

The Tiepie-HS5 incorporates two signal receiving channels and an arbitrary waveform generator. This generator can output waveform signals with frequencies ranging from 1 μHz to 40 MHz and a peak-to-peak voltage of 24 V. The excitation coil connects to the HS-5 through its Bayonet Nut Connector (BNC) interface, which supplies power to the excitation coil while collecting energy from the receiving coil through the HS-5’s receiving channels. During system operation, while the HS-5’s power output affects the amplitude of ultrasonic echo signals, it does not significantly impact their signal-to-noise ratio. Therefore, the excellent performance characteristics of the HS-5 make it suitable for our experimental requirements.

To facilitate WPT, we employed PCB coils as inductive elements in the WPT module. The coil parameters, such as the number of turns, were designed based on the simulation results of Section 2.2.2. The optimal coil parameters are shown in Table 1.

To simulate the WPT setup and control the lift-off height, we drilled holes in the PCB board to accommodate hexagonal spacers. The threaded length of these spacers was used to represent the lift-off height between the transmitting and receiving coils. This configuration allows us to precisely control and adjust the distance between the coils, mimicking various real-world scenarios of WPT.

To align our study with practical industrial applications, we selected aluminum and steel as the primary materials for our test specimens. These materials were chosen due to their widespread use in industrial equipment, ensuring that our experimental conditions closely mirror real-world scenarios.

We prepared a series of samples with the same dimensions of 150 mm × 150 mm, but with varying thicknesses of 6, 8, 10, 12, and 14 mm for both aluminum and steel. This range of thicknesses was selected to thoroughly evaluate the reliability and accuracy of our WPT-based ultrasonic thickness measurement system across different material thicknesses. Furthermore, samples of aluminum and steel pipes of the same thickness were prepared to study the effect of the shape.

## 3. Results and Discussion

### 3.1. Simulation Results

**Figure 8:**
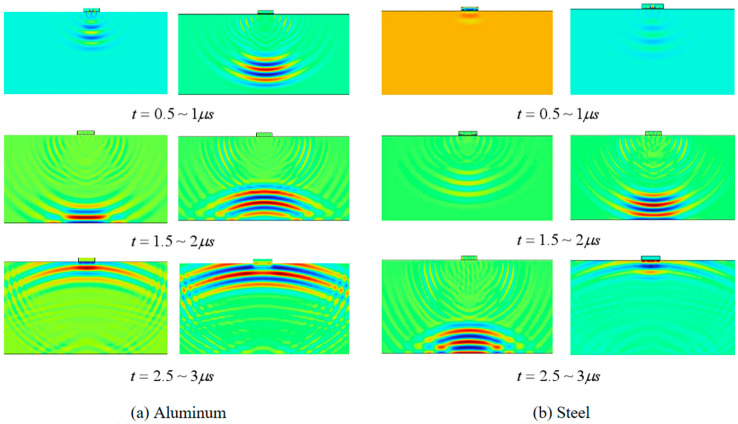
Simulation diagram of ultrasonic propagation in different metal plates at different times.

Figure 8 shows the entire process of the piezoelectric transducer converting electrical energy into mechanical energy and generating ultrasonic waves, which propagate through the matching layer to the aluminum plate and steel plate samples, respectively. It can be clearly seen that, after ultrasonic waves propagate to the bottom plate, most of the wave energy rebounds. In addition, the propagation speed of ultrasonic waves in aluminum plates is faster than that in steel plates, which matches the law of material acoustic impedance characteristics. The simulation implemented a five-cycle linear frequency modulated signal with window modulation. The excitation signal parameters were set to a center frequency of 5 MHz and a bandwidth (B) of 2 MHz. The mathematical expression for generating the excitation signal is given by the following:

(14) $$F(t)=\sin(2*\pi *f-\pi *B*t+\pi *\frac{B}{T}*t^{2})*0.5*(1-\cos(\frac{2*\pi *t}{T}))$$

**Figure 9:**
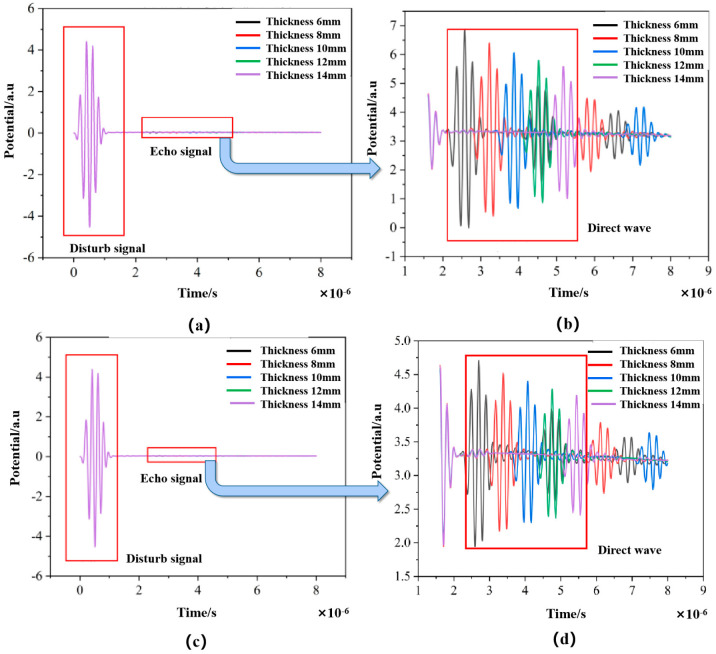
Transducer signal under aluminum plates of different thicknesses (**a**) before and (**b**) after removing crosstalk, and under steel plates of different thicknesses (**c**) before and (**d**) after removing crosstalk.

We conducted simulations of thickness measurements using the ultrasonic reflection method for both aluminum and steel plates of varying thicknesses. The simulation results are presented in Figure 9. Due to the presence of initial crosstalk, the bottom reflection waves cannot be clearly observed in Figure 9a,c. In order to obtain apparent echo signals, the crosstalk was set to zero, as shown in Figure 9b,d. The processed waveforms demonstrate that as the thickness of the specimen increases, the arrival time of the ultrasonic echo is correspondingly delayed.

**Figure 10:**
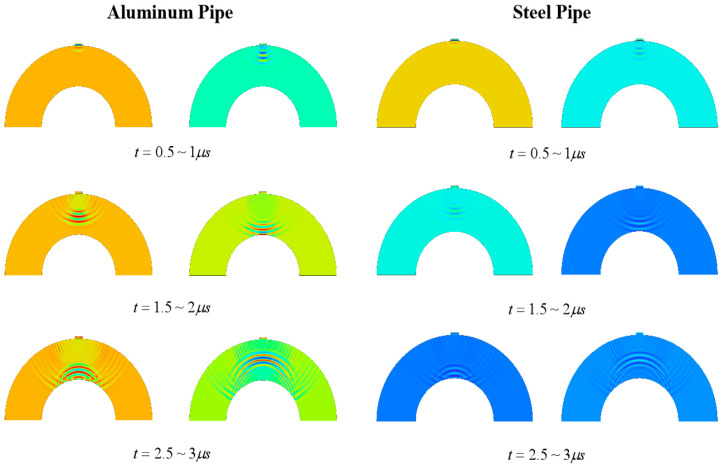
Simulation diagram of ultrasonic propagation in different metal tubes at different times.

Similarly to flat plate simulation, a simulation analysis of the ultrasonic wave propagation process in steel and aluminum pipes was conducted. The results are shown in Figure 10. In addition, a parameterized scanning simulation analysis of the pattern sizes of different metal pipe materials was also conducted to explore the propagation patterns of ultrasonic bulk waves in pipeline structures of different thicknesses. The results of the parameterized scanning are shown in Figure 10.

**Figure 11:**
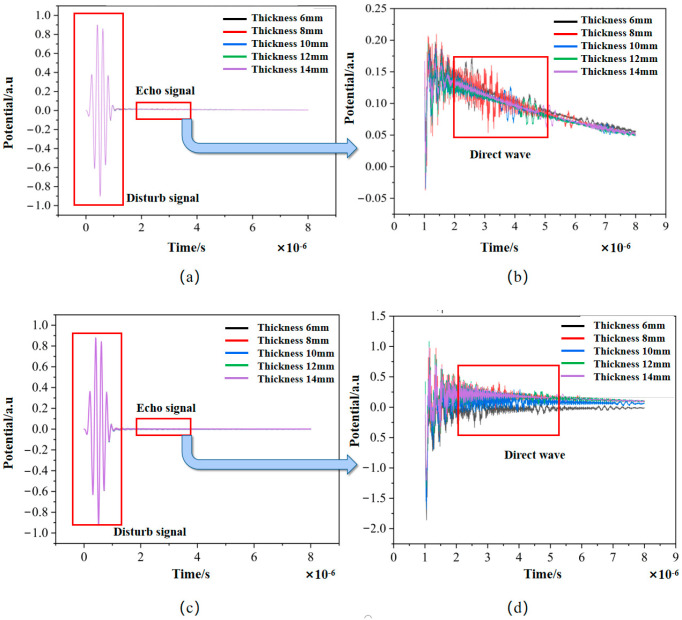
Transducer signal under aluminum pipes of different thicknesses (**a**) before and (**b**) after removing crosstalk and under steel pipes of different thicknesses (**c**) before and (**d**) after removing crosstalk.

As can be seen from Figure 10 and Figure 11, the echo signal generated after the excitation signal of the main wave becomes uneven, which also occurs in the potential after removing the crosstalk. The main reason for this phenomenon is that, due to the arc shape of the pipe, the ultrasonic wave continues to reflect within the matching layer when entering the pipe through the matching layer, leading to fluctuations at the end of the piezoelectric wafer, causing potential changes. When studying the propagation characteristics by extracting the transducer potential through parametric scanning, the reflected wave at the bottom boundary has a low signal-to-noise ratio, and the reflected wave at the bottom is submerged in noise. Therefore, when measuring the thickness of equipment, especially the thickness of pipes, signal processing on the ultrasonic thickness measurement signal is necessary to improve the accuracy of thickness measurement.

These simulations provide valuable insights into the behavior of ultrasonic waves in different materials and thicknesses, enabling us to refine our measurement algorithms and improve the accuracy of our wireless ultrasonic thickness measurement system.

### 3.2. Experimental Result

The experiment involved using a PC-controlled Tiepie-HS5 to generate a excitation signal. The excitation signal used was a linear frequency-modulated pulse signal with a center frequency of 5 MHz, a bandwidth of 2 MHz, and five cycles. It was also modulated by a window function. The pulse signal was then transmitted to power the piezoelectric transducers via a wireless energy transfer module. During the thickness measurement using the piezoelectric transducers, a coupling agent was applied between the test specimen and the piezoelectric transducer to enhance the transmission of ultrasonic waves generated by the transducer into the specimen. The voltage signals received back from the piezoelectric transducers through the wireless energy transfer module were captured by the Tiepie-HS5. Through the above methods, actual experiments were conducted on aluminum plates, steel plates, aluminum tubes, and steel tubes of different thicknesses, and pulse compression algorithms were used to denoise and filter the echo signals.

**Figure 12:**
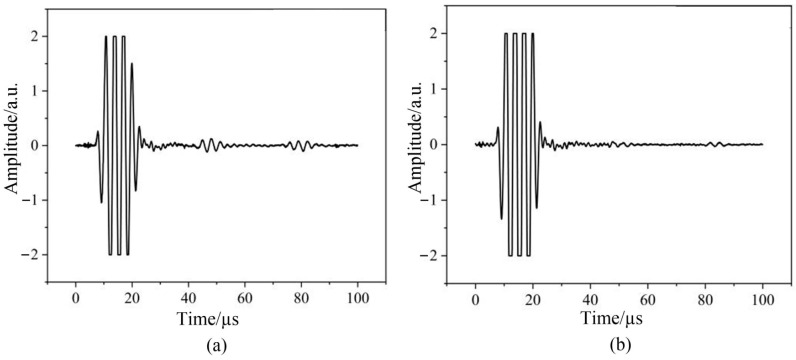
Transducer-received signals in different materials: (**a**) original received signal from a 10 mm aluminum plate; (**b**) original received signal from a 10 mm steel plate.

Figure 12 displays the extracted signals belonging to samples of different materials with a thickness of 10 mm. Due to the presence of front-end crosstalk, it is difficult to distinguish the ultrasonic echo signals and extract the peak time of the echo for thickness calculations. Therefore, a manual approach of setting the front-end crosstalk to zero was used to facilitate a more straightforward analysis of the ultrasonic echo signals in data processing. The processed data are shown in Figure 12.

**Figure 13:**
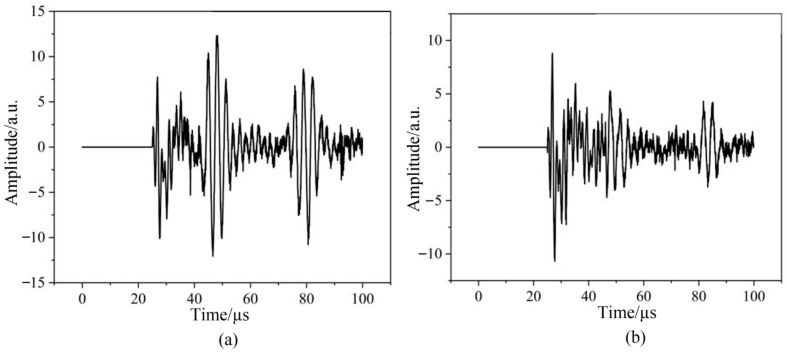
Echo signals from (**a**) a 10 mm aluminum plate and (**b**) a 10 mm steel plate after crosstalk removal.

As shown in Figure 12 and Figure 13, the signal-to-noise ratio of the echo signal is significantly enhanced after pulse compression algorithm processing, and the echo peak signal is clearer than before. After the envelope algorithm and peak time difference-solving algorithm are applied, the thickness value of the test sample can be accurately calculated, effectively improving the signal-to-noise ratio.

**Figure 14:**
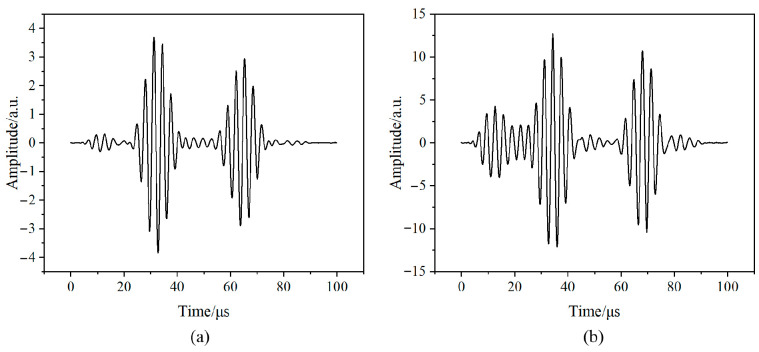
Pulse compression echo signals from (**a**) a 10 mm aluminum plate and (**b**) a 10 mm steel plate after crosstalk removal.

Based on the theory of pulse compression, the echo signal after removing crosstalk was analyzed using the pulse compression algorithm, and the results are shown in Figure 14.

**Figure 15:**
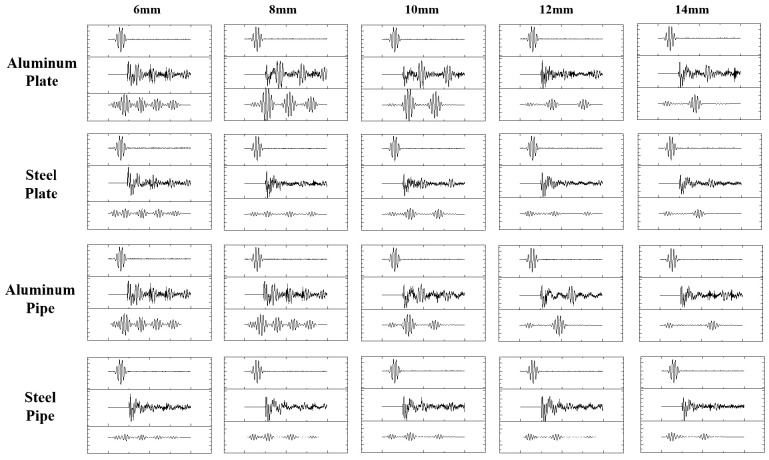
The results of wireless passive thickness measurement data of samples with different materials, shapes, and thicknesses processed by pulse compression algorithm.

The wireless passive thickness measurement data of aluminum plates, steel plates, aluminum tubes, and steel tubes with a thickness of 6–14 mm were processed by using pulse compression algorithm, and the results are shown in Figure 15. It can be seen that the pulse compression algorithm exhibits good applicability to the thickness measurement echo signals of samples with different parameters.

**Figure 16:**
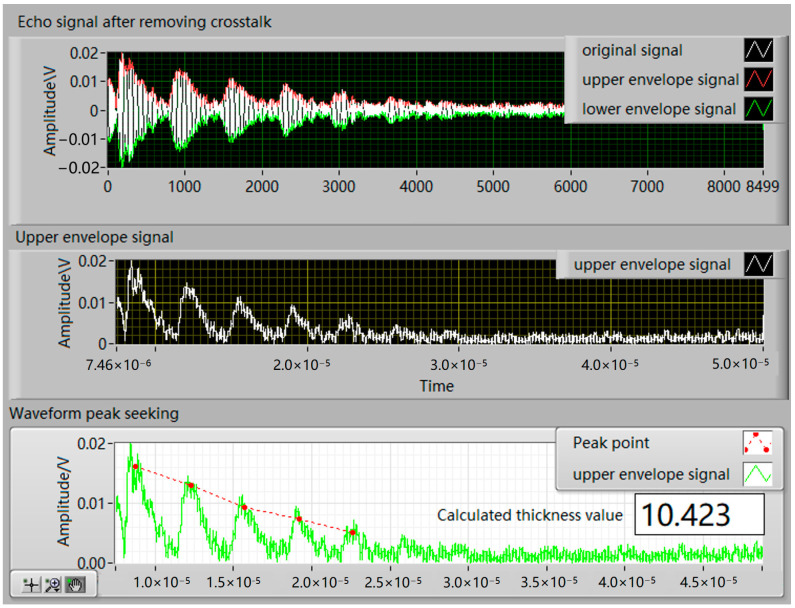
Screenshot of the upper computer interface for thickness measurement algorithm.

In order to verify the advantages of the pulse compression algorithm, thickness calculations were performed on both the compressed and unprocessed signals. By writing an upper computer program, the signal to be measured thickness was subjected to envelope processing and peak seeking processing, and then the thickness was calculated based on the peak time difference. The upper computer interface is shown in Figure 16.

As shown in the original signal waveforms in Figure 12 and Figure 14, the received ultrasonic signals exhibit poor signal-to-noise ratio due to the combined interference from wireless transmission and noise, causing the waveform signals to be submerged in noise. This noise interference significantly impacts the accuracy of envelope extraction and peak detection processes. When the pulse compression algorithm is applied to process the waveform signals, clean waveforms can be effectively extracted from the noise. Figure 14 demonstrates that the signal-to-noise ratio is substantially improved after pulse compression processing. Consequently, when using the time-of-flight method for thickness calculation, the measurement accuracy is significantly enhanced due to the precise detection of peak points. Additionally, since the thickness calculation also depends on the specimen’s sound velocity, obtaining more accurate sound velocity values through calibration would further reduce thickness measurement errors.

**Figure 17:**
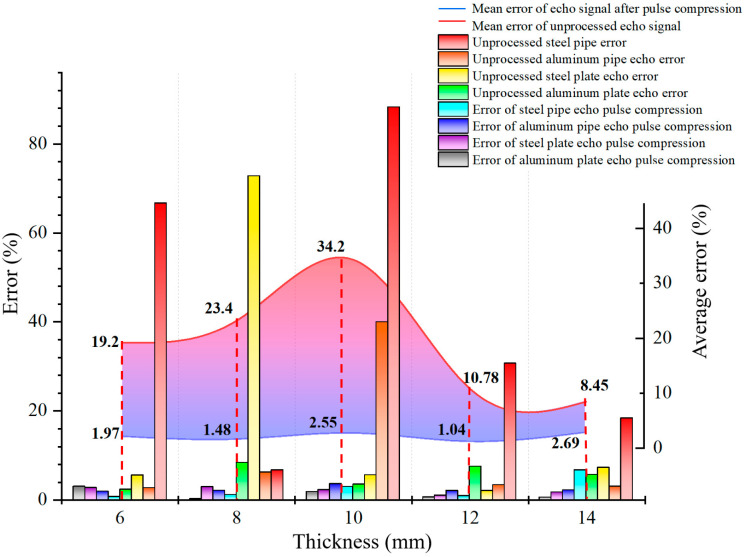
Comparison of thickness measurement results between pulse compressed echo signal and unprocessed echo signal.

The measurement error results are shown in Figure 17. It can be seen that direct thickness measurements of echo signals without pulse compression result in significant errors ranging from 8.45 to 34.2%; The detection accuracy of the echo signal after pulse compression is greatly improved, with an error range of 1.04–2.69%, and the comprehensive accuracy is increased by about 10 times.

The comparison result confirms the applicability of the pulse compression algorithm for wireless passive ultrasonic thickness measurement echo signals, providing important examples for the application of wireless passive ultrasonic thickness measurement technology in petrochemical sites.

## 4. Conclusions

This paper innovatively proposes a wireless passive ultrasonic thickness measurement technology that includes a pulse compression algorithm. Firstly, a mathematical model and equivalent circuit of single coil wireless energy transmission were constructed, and an implementation scheme for a three-terminal wireless energy mutual coupling system was designed, laying a solid foundation for energy transmission in subsequent ultrasound measurements. Secondly, a finite element analysis method was used to establish an ultrasonic body wave thickness measurement model, verifying the feasibility of the technology. Finally, an experimental system was built to conduct thickness measurement tests on metal samples with different sizes, shapes, and materials. The experimental results showed that, after introducing the pulse compression algorithm, the overall detection accuracy improved by approximately 10 times, with the error range effectively controlled between 1.04% and 2.69%. The experiment demonstrated the effectiveness of the pulse compression algorithm in optimizing the processing of wireless passive ultrasonic thickness measurement echo signals, and provided a valuable theoretical basis and practical guidance for the widespread application of this technology in industrial fields, particularly petrochemicals.

## Figures and Tables

**Table 1:** Three-coil design parameter table.

Key Parameter	Excitation Coil	Detection Coil	Receiving Coil
Number of turns	6	14	16
Outer diameter of coil	35 mm	20 mm	22 mm
Wire diameter	0.105 mm		
Line width	0.102 mm		
Relative magnetic permeability	1 N/A^2^		
Relative dielectric constant	4.4 F/m		

## Data Availability

The original contributions presented in the study are included in the article; further inquiries can be directed to the corresponding author.
